# Type VI Secretion System Accessory Protein TagAB-5 Promotes *Burkholderia pseudomallei* Pathogenicity in Human Microglia

**DOI:** 10.3390/biomedicines11112927

**Published:** 2023-10-30

**Authors:** Sanisa Lohitthai, Amporn Rungruengkitkun, Niramol Jitprasutwit, Thida Kong-Ngoen, Taksaon Duangurai, Sarunporn Tandhavanant, Passanesh Sukphopetch, Narisara Chantratita, Nitaya Indrawattana, Pornpan Pumirat

**Affiliations:** 1 Department of Microbiology and Immunology, Faculty of Tropical Medicine, Mahidol University, Bangkok 10400, Thailand; wipawee.loh@student.mahidol.edu (S.L.); amporn.run@mahidol.ac.th (A.R.); thida.kon@mahidol.ac.th (T.K.-N.); sarunporn.tan@mahidol.ac.th (S.T.); natthanej.lup@mahidol.ac.th (P.S.); narisara.cha@mahidol.ac.th (N.C.); nitaya.ind@mahidol.ac.th (N.I.); 2 Center for Vaccine Development, Institute of Molecular Biosciences, Mahidol University, Nakhon Pathom 73170, Thailand; niramol.jit@mahidol.edu; 3 Department of Companion Animal Clinical Sciences, Kasetsart University, Bangkok 10900, Thailand; taksaon.du@ku.th; 4 Department of Bacteriology, Institute of Tropical Medicine, Nagasaki University, Nagasaki 852-8523, Japan; 5 Mahidol-Oxford Tropical Medicine Research Unit, Faculty of Tropical Medicine, Mahidol University, Bangkok 10400, Thailand

**Keywords:** *Burkholderia pseudomallei*, neuropathogenesis, type VI secretion system, *Galleria mellonella*

## Abstract

Central nervous system (CNS) melioidosis caused by *Burkholderia pseudomallei* is being increasingly reported. Because of the high mortality associated with CNS melioidosis, understanding the underlying mechanism of *B. pseudomallei* pathogenesis in the CNS needs to be intensively investigated to develop better therapeutic strategies against this deadly disease. The type VI secretion system (T6SS) is a multiprotein machine that uses a spring-like mechanism to inject effectors into target cells to benefit the infection process. In this study, the role of the T6SS accessory protein TagAB-5 in *B. pseudomallei* pathogenicity was examined using the human microglial cell line HCM3, a unique resident immune cell of the CNS acting as a primary mediator of inflammation. We constructed *B. pseudomallei tagAB-5* mutant and complementary strains by the markerless allele replacement method. The effects of *tagAB-5* deletion on the pathogenicity of *B. pseudomallei* were studied by bacterial infection assays of HCM3 cells. Compared with the wild type, the *tagAB-5* mutant exhibited defective pathogenic abilities in intracellular replication, multinucleated giant cell formation, and induction of cell damage. Additionally, infection by the *tagAB-5* mutant elicited a decreased production of interleukin 8 (IL-8) in HCM3, suggesting that efficient pathogenicity of *B. pseudomallei* is required for IL-8 production in microglia. However, no significant differences in virulence in the *Galleria mellonella* model were observed between the *tagAB-5* mutant and the wild type. Taken together, this study indicated that microglia might be an important intracellular niche for *B. pseudomallei,* particularly in CNS infection, and TagAB-5 confers *B. pseudomallei* pathogenicity in these cells.

## 1. Introduction

*Burkholderia pseudomallei* is a Gram-negative pathogen that is responsible for melioidosis, which is endemic to areas of Southeast Asia, Northern Queensland, and the Northern Territory of Australia [1,2]. Additionally, there is increasing evidence that melioidosis exists in other areas, including the United States [3] and Bangladesh [4]. This disease is considered a great mimicker as it presents with a varied range of disease manifestations. Although the clinical symptoms commonly present as a lung infection, septicemia, and multiple internal abscesses; because of *B. pseudomallei* pathogenicity, it is able to affect any vital organs in the body, including the central nervous system (CNS). Melioidosis of the CNS accounts for approximately 1.5–3.0% of all melioidosis cases [5,6]. CNS melioidosis has varying presentations that include symptoms resembling Guillain–Barré syndrome, limb weakness, and cranial nerve palsies [7,8]. CNS melioidosis is of significant importance because it has a mortality of approximately 25% and survivors have significant morbidity [9,10,11]. Moreover, CNS melioidosis has been increasingly reported in recent years [8]. Therefore, neuropathogenesis of CNS melioidosis has received more attention.

*B. pseudomallei* can penetrate the CNS via the olfactory and trigeminal nerves within the nasal cavity [9,10]. One study reported that *B. pseudomallei* use actin-based motility to facilitate initial infection of the olfactory epithelium and penetrate the brain stem and spinal cord [10]. Furthermore, another study proposed that neurological infection arises through infected monocytes that serve as a Trojan horse and migrate across the blood–brain barrier to destroy neural tissue [12,13]. In the CNS, the main components of the nervous system comprise neurons and glial and microglial cells [14,15]. Neurons are responsible for detecting change and communicating with other neurons. Glial cells serve physical and chemical support to neurons and maintains their environment, while microglia are the key innate immune effector cells of the CNS. Currently, there are still few reports on the molecular mechanism underlying *B. pseudomallei* and CNS cell interaction. In vitro study of *B. pseudomallei* pathogenicity in human neuronal SH-SY5Y cells revealed that it employs a virulence factor called “cycle inhibiting factor” to invade human neuronal SH-SY5Y cells [16]. A recent study has shown that *Burkholderia* intracellular motility A (BimA) is required for successful intracellular survival, multinucleated giant cell (MNGC) formation, and induction of cytotoxicity and apoptosis of human neuronal cells [17]. However, there is no information regarding CNS cell immune defense in response to *B. pseudomallei* infection.

In this study, we focused on the investigation of *B. pseudomallei* neuropathogenesis in microglial cells, the resident macrophages of the CNS. Similar to other macrophages, microglial cells work as innate immune cells in the CNS through phagocytosis and sterilization of foreign substances such as bacteria and play a central role in defending the host from infection. Microglial defense involves production of cytokines that are responsible for the early control of infections and for the recruitment of cells of the adaptive immune system required for pathogen clearance [18]. Microglial activation results in their production of proinflammatory cytokines such as interleukin 1 (IL-1), interleukin 6 (IL-6), interleukin 8 (IL-8), and tumor necrosis factor alpha (TNF-α) [19]. During murine melioidosis, the activation and expansion of microglia have been found in the brain [20]. However, the role of microglia against *B. pseudomallei* infection is still unknown.

It is evident that *B. pseudomallei* uses a type VI secretion system (T6SS) during macrophage infection [21,22]. The T6SS is a membrane complex formed by multiple protein components that uses a spring-like mechanism to inject effectors into target cells. Based on in vivo expression technology, a T6SS cluster 5 (T6SS-5, also termed T6SS1) was induced during *B. pseudomallei* invasion into the macrophages. Furthermore, upon vacuolar escape into the cytoplasm, *B. pseudomallei* manipulate host cells by utilizing the T6SS1 to form MNGC for intercellular spread [23]. Interestingly, one study revealed that lack of an accessory protein of T6SS TagAB-5 (BPSS1504) in *B. pseudomallei* impaired intracellular replication and MNGC formation in the murine macrophage RAW 264.7 cell line and attenuated them in a BALB/c mice model when compared with wild-type bacteria [21]. Therefore, our curiosity has driven us to explore the role of this accessory protein and its importance in pathogenesis in human microglial infection.

To examine the role of the TagAB-5 protein in human microglial infection, the *B. pseudomallei tagAB-5* mutant and complementary strains were constructed and tested with the human glial cell line HCM3 for internalization, intracellular survival, cytotoxicity induction, and MNGC formation. Cytokine production by HCM3 cells infected with these constructed *B. pseudomallei* strains was measured to investigate the pattern of cytokine induction. In addition, the virulence of the *B. pseudomallei tagAB-5* mutant strain was evaluated using *Galleria mellonella*, an in vivo model that has been used for studying brain infection by meningitis-causing bacteria [24]. Our findings have significant implications for understanding melioidosis, focusing on the molecular mechanisms of interaction between *B. pseudomallei* and CNS immune cells. Furthermore, this fundamental knowledge provides valuable insights for the development of novel therapeutic strategies to combat this lethal disease.

## 2. Materials and Methods

### 2.1. Biosecurity Aspects

General bacterial laboratory procedures were conducted in compliance with the security and safety regulations of Mahidol University in an enhanced BSL2 facility equipped with standard procedures for the BSL3 laboratory. This project has been approved by Mahidol University Institutional biosafety committee (Reference No: MU 2023-028).

### 2.2. Bacterial Strains, Cell Line, and Growth Conditions

*B. pseudomallei* strain K96243 was obtained from the previous study [25]. The *B. pseudomallei tagAB-5* deletion mutant and complementary strains were constructed in this study (Table 1). All bacterial strains were cultured in Luria–Burtani medium (LB; Difco Laboratory, Sparks, MD, USA) at 37 °C with shaking. The human microglial HMC3 cell line (ATCC^®^ CRL-3304™) was maintained in Eagle’s Minimum Essential Medium (EMEM; ATCC) supplemented with 10% (*v*/*v*) heat-inactivated fetal bovine serum (FBS; Gibco BRL, Billings, MT, USA) and penicillin-streptomycin solution (Gibco BRL) at 37 °C in a humidity-controlled incubator with 5% CO_2_. The cell culture medium was replaced with fresh medium every other day. The confluent cells were harvested with 0.25% (*w*/*v*) trypsin-EDTA solution (Gibco).

### 2.3. Construction of B. pseudomallei tagAB-5 Deletion Mutant and Complementary Strains

The *B. pseudomallei tagAB-5* mutant and complementary strains were constructed using the markerless allele replacement method [26]. To delete the *tagAB-5* gene, a DNA fragment consisting of 650 bp upstream and 536 bp downstream regions, derived from GenBank (locus_tag = “BPSS1504”) of *B. pseudomallei* K96243, was synthesized and cloned into the pUC57 vector (GenScript). Subsequently, the fragment was released through digestion with *Not*I and *Eco*RI (New England Biolabs), followed by ligation to the pEXKm5 vector using the same restriction enzymes. The resulting plasmid was transformed into *E. coli* RHO3 and mobilized into *B. pseudomallei* K96243 using the conjugation method. The obtained conjugants were selected on LB agar containing 1000 μg/mL of kanamycin and incubated for 24–48 h at 37 °C. To resolve the merodiploid, the bacteria were grown on yeast extract-tryptone agar containing 15% (*w*/*v*) sucrose and screened for kanamycin-sensitive clones. The *tagAB-5* deletion mutants were validated by PCR using primers flanking the deleted alleles, Seq-F-BPSS1504 and Seq-R-BPSS1504 (Table 2). Additionally, the absence of the pEXKm5 plasmid was confirmed using *oriT* primers (Table 2) [16].

To facilitate gene complementation, a similar pEXKm5-based allele exchange method was utilized. The full-length sequence of *B. pseudomallei* K96243 *tagAB-5* was amplified using the F1-BPSS1504 and R2-BPSS1504 primers (Table 2). Then, the resulting DNA product was subjected to *Sma*I digestion and subsequently ligated with the *Sma*I-digested pEXKm5 vector. Similar to the generation of the deletion mutant, the pEXKm5 containing the whole sequence of *tagAB-5* was transformed into *E. coli* RHO3 for conjugation into the *B. pseudomallei tagAB-5* mutant. The complementation of *tagAB-5* was screened by PCR using primers flanking the deleted alleles, Seq-F-BPSS1504 and Seq-R-BPSS1504 (Table 2) and verified by DNA sequencing. Schemes of recombinant constructions were created using a molecular biology software (version 7.0, SnapGene, San Diego, CA, USA).

### 2.4. Growth Assay

A single isolated colony of *B. pseudomallei* was inoculated in LB broth and incubated at 37 °C with shaking at 200 rpm for 24 h. Then, the overnight-culture of bacteria was washed with phosphate buffered saline (PBS) and adjusted to an optical density (OD) at 600 nm (OD_600_) of 0.5. To examine the growth kinetics, the prepared *B. pseudomallei* was added into fresh LB medium at a ratio of 1:500 and incubated at 37 °C with shaking at 200 rpm. OD_600_ was measured at predetermined time points. For colony morphology, *B. pseudomallei* on Ashdown’s agar at Day 4 was examined using a morphotyping algorithm [27].

### 2.5. Internalization and Intracellular Replication Assay

Human microglial HMC3 cells were seeded at a density of 5 × 10^4^ cells per well in a 24-well cell culture plate. The next day, the medium was removed and replaced with 200 µL of fresh antibiotic-free EMEM. Overnight cultures of *B. pseudomallei* strains were adjusted to 1 × 10^6^ cells per ml by OD measurement at 600 nm and used to infect the cells at the required multiplicity of infection (MOI). After 2 h of co-culturing, the infected cells were washed twice with PBS, and then 500 µL of fresh EMEM containing 250 µg/mL kanamycin (Sigma-Aldrich, Saint Louis, MO, USA) was added and incubated at 37 °C for 1 h to eliminate any extracellular bacteria. To recover the internalized bacteria, *B. pseudomallei*-infected HMC3 cells were washed twice with PBS before cell lysis with 0.1% (*w*/*v*) Triton X-100. The number of viable bacteria was determined as colony forming units (CFUs) by a serial dilution of bacterial culture, with 10 µL of each dilution dropped on LB agar and incubated at 37 °C for 24 h. Similarly, the replication assay was performed at 4, 6, 8, and 10 h post-infection to assess intracellular number of viable *B. pseudomallei* strains in human HMC3 cells. Doubling time was calculated by dividing the natural logarithm of 2 by the exponent of bacterial growth rate [28], calculated using the following equations.

b = B × 2^n^

n = 3.3 logb/B

where B = number of bacteria at the beginning of a time interval, b = number of bacteria at the end of the time interval, n = number of generations

$$k=\frac{n}{t}=\frac{\log N_{1}-\;\log N_{0}}{\log2}=\frac{\log N_{1}-\;\log N_{0}}{0.30t}$$

where, k = the growth rate constant, $N_{0}$ = the initial population at the time 0, $N_{1}$ = the population at the time t, n = the number of generations in time t

$$\mathrm{Doubling}\;\mathrm{time}=\frac{\log(2)}{\log(1+r)}$$

where r = the growth rate constant.

### 2.6. Multinucleated Giant Cell (MNGC) Formation Assay

MNGC was determined as described previously with some modifications [16]. Briefly, 5 × 10^4^ HMC3 cells were grown on coverslips in 500 µL of fresh EMEM in 24-well cell culture plate at 37 °C with 5% CO_2_ overnight. The next day, the medium was replaced with 200 µL of fresh antibiotic-free EMEM. HMC3 cells were infected with 1 × 10^6^ cells per ml of *B. pseudomallei* to obtain an MOI of 2 and then incubated at 37 °C with 5% CO_2_ for 2 h. The infected cells were washed twice with PBS, and then 500 µL of fresh EMEM containing 250 µg/mL kanamycin (Sigma-Aldrich, Saint Louis, MO, USA) was added to eliminate any extracellular bacteria. At 8 h post-infection, infected HMC3 cells were washed with PBS and fixed with 4% (*w*/*v*) of paraformaldehyde in PBS overnight at room temperature. Then, the HMC3 cells were washed and covered with 50% (*v*/*v*) and 90% (*v*/*v*) ethanol, respectively. Then, the HMC3 cells were stained with Giemsa stain (Merck, Darmstadt, Germany) for 5 min, rinsed in distilled water, and air-dried.

MNGCs were defined as cells containing at least three nuclei. MNGC formation efficiency (as a percentage) was determined with a 40× objective using the following formula: (N within multinucleated giant cells/total N) × 100, where N is the number of nuclei.

### 2.7. Actin Tail Formation Assay

The microglial HMC3 cells were seeded on 12-mm round glass coverslips (Menzel-Gläser, Braunschweig, Germany) of fresh EMEM in a 24-well plate (Costar, Corning, NY, USA) and incubated at 37 °C in a humidified 5% CO_2_ atmosphere before the infection. The overnight *B. pseudomallei* strain was subjected to infection at an MOI of 2 after the medium was replaced with 200 µL of fresh antibiotic-free EMEM. The infected cells were incubated at 37 °C with 5% CO_2_ for 2 h and then were washed by PBS twice. After that, extracellular bacteria were killed by adding 500 µL of fresh EMEM containing 250 µg/mL kanamycin (Sigma-Aldrich, Saint Louis, MO, USA). At 8 h post-infection, the infected cells were washed with PBS and fixed with 4% (*v*/*v*) paraformaldehyde in PBS for 24 h. The fixed cells were washed with PBS before permeabilization with 0.5% (*v*/*v*) Triton X-100 in PBS for 30 min, then 1% (*w*/*v*) bovine serum albumin (Sigma-Aldrich, Saint Louis, MO, USA) in PBS was added and the cells were incubated for 30 min at room temperature. Subsequently, bacteria were stained using a mouse monoclonal anti-*B. pseudomallei* lipopolysaccharide antibody (Camlab, Cambridge, United Kingdom), followed by Alexa Fluor555-conjugated anti-mouse immunoglobulin (Molecular Probes, Eugene, OR, USA). Actin filaments and DNA were stained using Alexa Fluor488-conjugated phalloidin (Molecular Probes) and ProLong Gold antifade with DAPI (Invitrogen, Eugene, OR, USA), respectively. Actin-tail formation was observed in 100 fields by confocal laser scanning microscopy (LSM 700; Carl Zeiss, Jena, Germany).

### 2.8. Lactate Dehydrogenase (LDH) Detection

The microglial HCM3 cells were seeded at 5 × 10^4^ cells per well in a 24-well cell culture plate and infected with *B. pseudomallei* at an MOI of 2 for 10 h. Then, the supernatants of infecting media were collected and filtered using a 0.22-μm filter. The CytoTox96 kit (Promega, Madison, WI, USA) was used according to the manufacturer’s instructions. In brief, the mixture of filtered supernatant and CytoTox 96 reagent was incubated in the ELISA plate at room temperature for 30 min. Then, the stop solution was added and the optical density at 490 nm (OD_490_) determined by an ELISA reader (Tecan Sunrise microplate reader). The percentage of cytotoxicity from LDH released was calculated by using the following equation: (OD_490_ experimental release—OD_490_ spontaneous release)/(OD_490_ maximum release—OD_490_ spontaneous release) × 100. The amount of LDH released from uninfected cells were considered as a spontaneous release, whereas the maximum release of LDH was obtained by lysing uninfected cells with 0.1% (*v*/*v*) Triton X-100.

### 2.9. Cytokine Expression

The IL-8 and TNF-α released from *B. pseudomallei*-infected-HMC3 cells was determined by using human IL-8 and TNF-α–enzyme-linked immunosorbent assay (ELISA) kit, (Abcam, Cambridge, United Kingdom), as per the manufacturer’s instructions. Briefly, 50 μL of each supernatant sample was added in triplicate to a designed well, which was an antibody capture coated well. Then, 50 µL was added to the reaction well with 50 µL of working detector and the samples were incubated at room temperature for 1 h with shaking at 400 rpm, following by washing with washing buffer for 3 times before adding 100 µL of tetramethylbenzidine (TMB) as a substrate reagent, and then samples were incubated at room temperature for 10 min. After that, 100 µL of stop solution was added. The mixture was mixed by shaking the ELISA plate for 1 min. The absorbance at 450 nm was measured by a Sunrise^TM^ Absorbance Reader (Tecan, Männedorf, Switzerland). The standard curve was performed coupled with each assay.

### 2.10. Antimicrobial Susceptibility Testing

*B. pseudomallei* strains were determined for antimicrobial susceptibility using the Kirby–Bauer disc diffusion method according to instruction of European Committee on Antimicrobial Susceptibility Testing (EUCAST) [29]. The following antibiotics were tested: amoxicillin-clavulanic acid (AMC, 30 µg), ceftazidime (CAZ, 30 µg), imipenem (IMP, 10 µg), meropenem (MEM, 10 µg), trimethoprim-sulfamethazole (SXT, 25 µg), tetracycline (TE, 30 µg), and chloramphenicol (C, 30 µg). Briefly, a 0.5 McFarland suspension of *B. pseudomallei* was prepared in normal saline and inoculated onto Mueller–Hinton agar (Oxoid Ltd., Basingstoke, Hampshire, UK). The antibiotic discs were placed at a specific distance from each other on the agar, and the zone of inhibition around each antibiotic disc was measured after 18–24 h of incubation at 37 °C. The zone of inhibition was interpreted as sensitive, intermediate, or resistant according to EUCAST guideline.

### 2.11. Galleria mellonella Killing Assay

*G. mellonella* killing assays were performed as previously described [30]. Fifty larvae were used in this experiment. All were 2–2.5 cm in length, 250–300 mg in body weight, and free of melanization. After 18 h of growth, *B. pseudomallei* was diluted to a concentration of 100 CFUs/mL in PBS by adjusting the OD_600_. A Hamilton syringe was used to inject 1 CFU of the bacterial suspension into the body cavity of *G. mellonella* larvae via the proleg. Each control larva was injected with PBS. Following injection, larvae was incubated in the dark at 37 °C. At 24, 30, 36, and 40 h post-injection, larvae were individually investigated for pigmentation and mobility. Larvae were considered dead when they displayed no movement in response to gentle prodding with a pipette tip. The numbers of dead larvae and times of death were recorded, and the survival graph was plotted.

### 2.12. Statistical Analysis

All assays were conducted in triplicate, and an unpaired *t*-test of two independent experiments was performed using the GraphPad Prism 8 (GraphPad Software, San Diego, CA, USA). Typically, only the wild-type and knockout strains were compared statistically. The complement strain was utilized for effect verification. Consequently, two sample tests were performed without requiring familywise error correction. Results were considered significant at a *p* value ≤ 0.05. For analysis of MNGC formation of HMC3 cells infected by *B. pseudomallei*, the Gaussian nature and equality of variances were also verified by the Shapiro–Wilk test and the F test, respectively. For the *G. mellonella* killing assay, a log-rank (Mantel–Cox) test by GraphPad Prism 8 was used to compare survival curves.

## 3. Results

### 3.1. Effect of TagAB-5 Deletion on B. pseudomallei Growth, Colony Morphology, and Antimicrobial Susceptibility

To elucidate the neuropathogenesis of the TagAB-5 T6SS accessory protein in human microglia, a *B. pseudomallei* mutant lacking the *tagAB-5* gene (Δ*tagAB-5*) and a complement strain (Δ*tagAB-5*::*tagAB-5*) were generated. The successful gene manipulation of all constructed strains was verified using PCR with specific primers (Figure S1). As expected, a 1349 bp DNA product (lane 4) was detected in the Δ*tagAB-5* mutant, indicating successful homologous recombination resulting in the deletion of 2660 bp of the *tagAB-5* gene from the chromosome of *B. pseudomallei*. The Δ*tagAB-5*::*tagAB-5* complement strain was constructed using the same approach, and the amplified product of the complement strain showed the presence of a 4009 bp DNA fragment (lane 5), similar to the parent strain.

**Figure 1:**
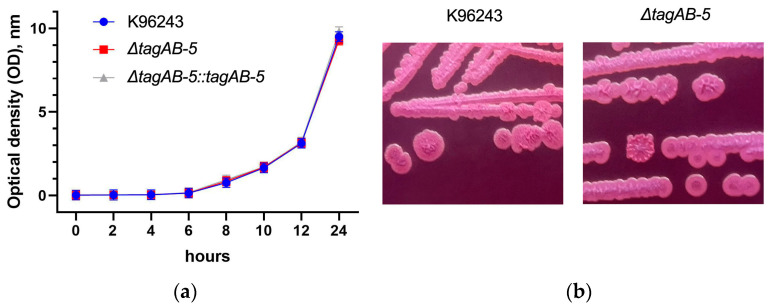
Growth character of *B. pseudomallei* stains used in this study. (**a**) *B. pseudomallei* K96243, Δ*tagAB-5*, and Δ*tagAB-5*::*tagAB-5* strains were grown in LB broth at 37 °C with shaking. OD was determined at 600 nm. The data points and error bars represent the mean ± SEM from triplicate experiments. (**b**) *B. pseudomallei* K96243 and Δ*tagAB-5* mutant growth on Ashdown’s agar for 4 days. The colony morphology was examined using a morphotyping algorithm.

To assess the impact of *tagAB-5* deletion on *B. pseudomallei* fitness, the growth rates of the Δ*tagAB-5* mutant and wild-type K96243 strain were compared in LB medium. As shown in Figure 1a, no significant differences were observed among these strains, including Δ*tagAB-5*::*tagAB-5* complement strain, indicating that the deletion of *tagAB-5* had no influence on the in vitro growth of *B. pseudomallei*.

Furthermore, the colony morphology and antimicrobial susceptibility of *B. pseudomallei* were examined to assess the effects of *tagAB-5* deletion. Morphotypes on Ashdown’s agar were divided into seven types (denoted I to VII) [27]. Type I is common morphotype that is able to transition to other morphotypes (most commonly type II or III) by a process of switching in response to environmental stress [27]. The result showed that both wild-type and mutant colonies exhibited the type I morphotype, with no significant differences observed (Figure 1b). Similarly, the antibiotic susceptibility profiles were identical between the wild-type and mutant (Table 3).

### 3.2. TagAB-5 Plays Role in Intracellular Survival of Human Microglia Cells

The range of MOIs for co-culturing of *B. pseudomallei* and HMC3 was determined to identify the most appropriate MOI for study of *B. pseudomallei* intracellular survival assay. From the result obtained, no bacteria were observed at 3 h post-infection using the MOI of 0.05, and viable intracellular bacteria were recovered from all tested MOIs of 0.05, 0.5, 1, 2, and 20 were detected at 4, 6, 8, and 10 h post-infection (Figure S2). Although the MOI of 20 provided the maximum number of intracellular bacteria during infection, this MOI resulted in the destruction of cellular monolayer due to the extensive damage of infected cells. While using MOI of 0.5, 1, and 2 were adequate to assess the intracellular survival capability of *B. pseudomallei* in HMC3 cells, the number of intracellular bacteria were increased when using a higher MOI. Additionally, the number of bacteria recovered from infected HMC3 at 10 h post-infection was significantly higher (*p* < 0.05) when using an MOI of 2, compared to using the MOI of 0.5 or 1. Therefore, the MOI of 2 was chosen for further experiments in this study.

**Figure 2:**
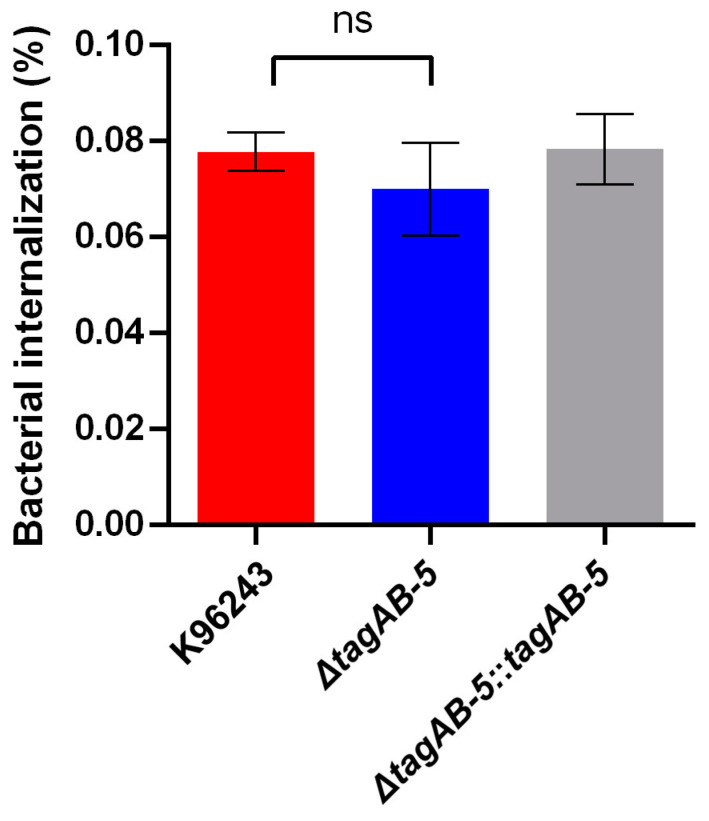
Internalization of *B. pseudomallei* into HCM3 cells. HMC3 cells were infected with *B. pseudomallei* K96243, Δ*tagAB-5*, and Δ*tagAB-5*::*tagAB-5* strains at an MOI of 2. The internalized bacteria into the HMC3 were recovered at 3 h post-infection. Values are shown as the mean ± SEM of three independent experiments. ns: not significant.

To determine the roles of the TagAB-5 in the intracellular survival of *B. pseudomallei* in human microglia. Co-culture of HMC3 cells and *B. pseudomallei* strains in this study was then investigated at an MOI of 2 for 10 h. At 3 h post-infection, the percentage of internalization of *B. pseudomallei* wild-type K96243, Δ*tagAB-5* mutant, and complemented Δ*tagAB-5*::*tagAB-5* strains was 0.078 ± 0.009%, 0.070 ± 0.010%, 0.078 ± 0.007%, respectively (Figure 2). The Δ*tagAB-5* mutant showed an internalization level similar to that of the wild-type strain. This result indicates that *tagAB-5* was not associated with the invasion ability of *B. pseudomallei* in HMC3 cells.

**Figure 3:**
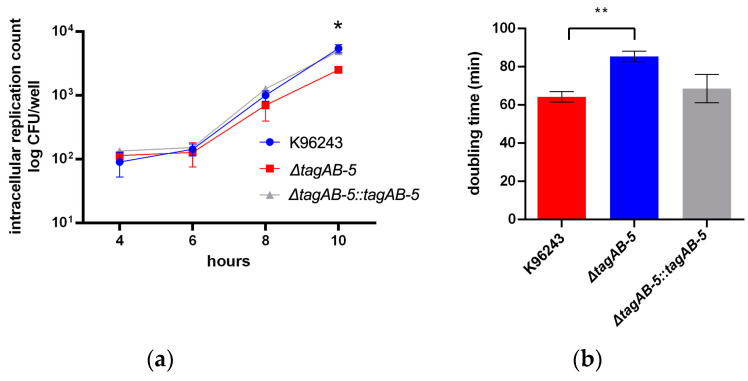
Intracellular survival of *B. pseudomallei* (**a**) HMC3 cells were infected with different strains of *B. pseudomallei* K96243, Δ*tagAB-5*, and Δ*tagAB-5*::*tagAB-5* at an MOI of 2. Intracellular multiplication was determined at 4, 6, 8, and 10 h post-infection. (**b**) Doubling time of *B. pseudomallei* in HMC3 cells at 10 h post-infection. Values are shown as the mean ± SEM of three independent experiments. * *p* < 0.05, ** *p* < 0.01.

With respect to intracellular survival, the intracellular bacteria were recovered by plating on culture medium plates at 4, 6, 8, and 10 h post-infection. There were no significant differences of this ability among the strains used in this study within 8 h after infection (Figure 3a). Until 10 h post-infection, Δ*tagAB-5* showed a statistically significant decrease in CFU when compared with the wild-type K96243 (*p* = 0.0219). Complementation of Δ*tagAB-5* can restore this phenotype. These results indicated that deletion of Δ*tagAB-5* can result in decreased intracellular survival ability in HMC3 cells (Figure 3a). Moreover, the doubling time of *B. pseudomallei* in HMC3 showed that the Δ*tagAB-5* was significantly higher than the wild-type strain K96243 (*p* = 0.0057), which were 85.4 ± 2.8 min and 64.3 ± 2.7 min, respectively (Figure 3b). This result suggests that TagAB-5 is involved with intracellular replication of *B. pseudomallei* in HMC3 cells.

### 3.3. TagAB-5 Is Required for Multinucleated Giant Cell (MNGC) Formation

**Figure 4:**
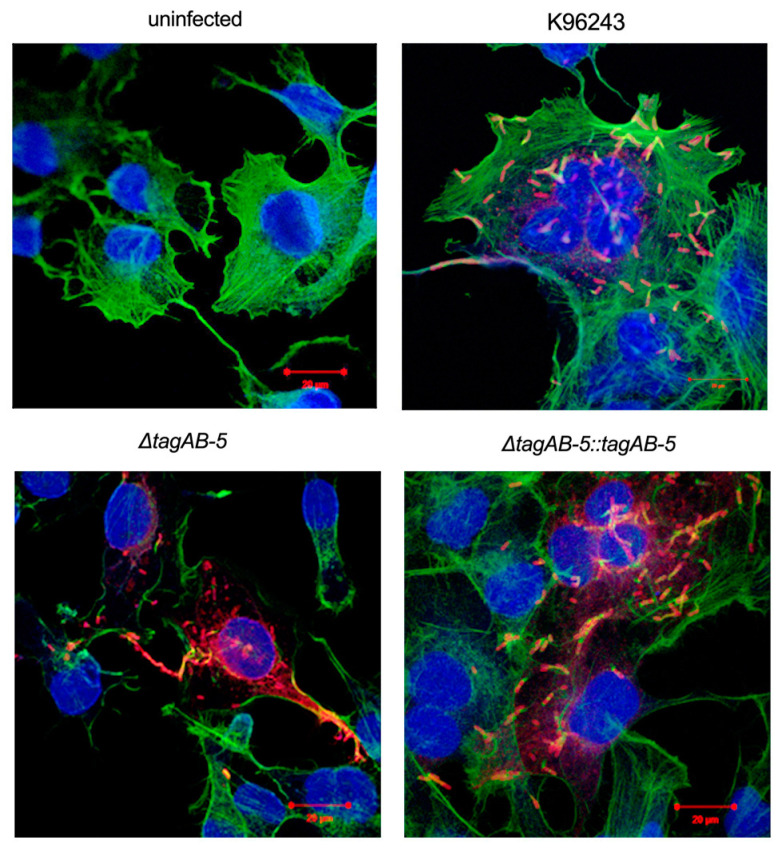
Actin tails of *B. pseudomallei* strains in HMC3 cells. HMC3 cells infected with *B. pseudomallei* K96243, Δ*tagAB-5*, and Δ*tagAB-5::tagAB-5* strains. At 8 h post-infection, the infected cells were stained to detect actin tails. Actin tails in HCM3 cells were examined by indirect immunofluorescence staining with Alexa Fluor488-conjugated goat anti-mouse IgG phalloidin (green), and DNA in nuclei was stained using DAPI (blue). Bacteria were stained using mouse monoclonal anti-*B. pseudomallei* lipopolysaccharide antibody and detected with Alexa Fluor555-conjugated phalloidin (red). Scale bar = 20 µm.

*B. pseudomallei* harbors actin-based motility for intra- and inter-cellular movement, leading to cell fusion for intracellular persistence without exposure to antimicrobial peptides or antibodies outside the cells. We examined whether the deletion of *tagAB-5* gene affect the formation of the actin tail of *B. pseudomallei* in microglial HCM3 cells. The result showed that the *tagAB-5*-deleted mutant harbors actin tails in HCM3 cells with a typical comet-tail phenotype (Figure 4). No differences in the actin-based motility of *B. pseudomallei* K96243, Δ*tagAB-5*, and Δ*tagAB-5*::*tagAB-5* upon HCM3 infection.

**Figure 5:**
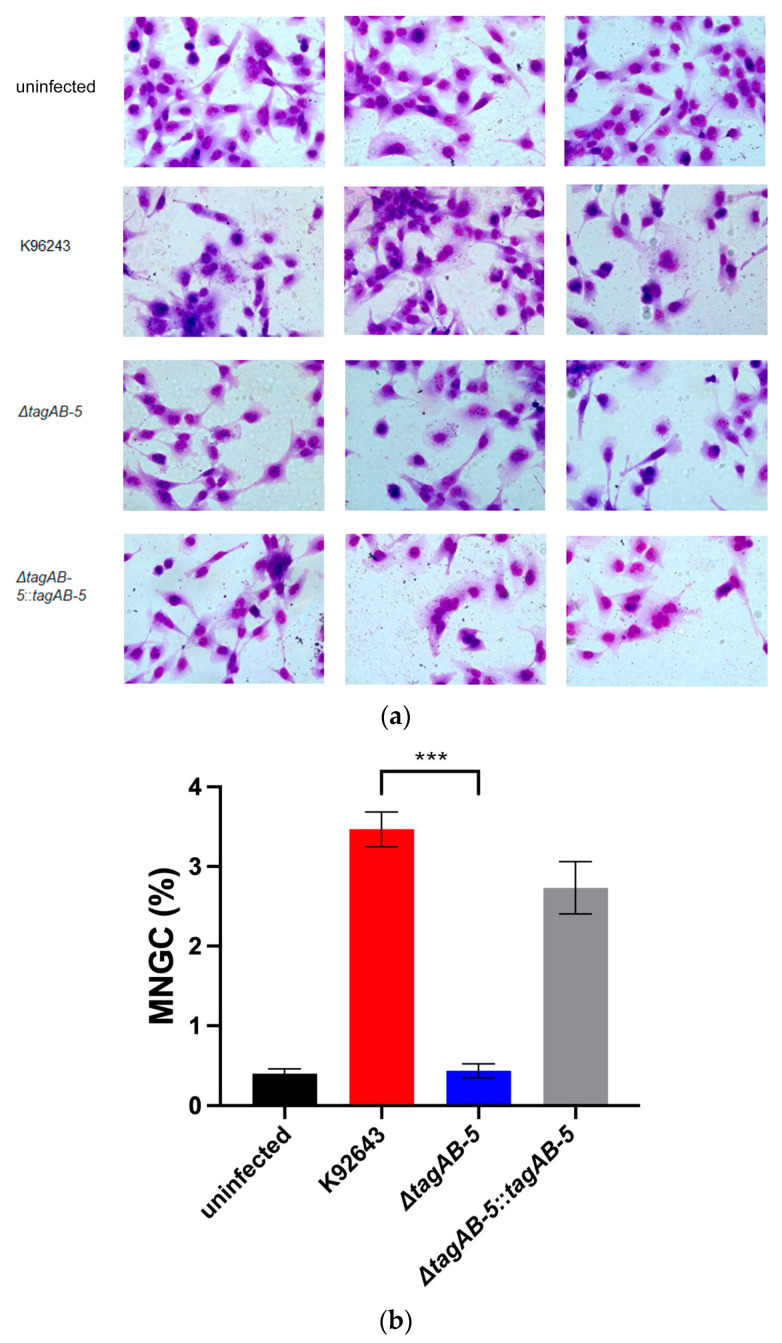
MNGC formation of HMC3 cells infected by *B. pseudomallei*. (**a**) HMC3 cells were co-cultured with *B. pseudomallei* K96243, Δ*tagAB-5*, and Δ*tagAB-5*::*tagAB-5* strains for 8 h. Cells were stained with Giemsa. Images were captured by light microscope (Zeiss; Axio Imager.M2) under a 40× objective lens. (**b**) The percentage of MNGC formation induced by *B. pseudomallei* strains. Values are shown as the mean ± SEM of three independent experiments. The Gaussian nature and equality of variances were verified by the Shapiro–Wilk test (*p*-value > 0.05) and the F test (*p*-value = 0.280), respectively. An unpaired t test was then used to compare means between the wild-type and knockout strains. *** *p* < 0.001.

Prominently, the unique characteristic of *B. pseudomallei* is cell-to-cell fusion leading to MNGC formation. Ability to form MNGCs has been previously reported to be involved with T6SS of *B. pseudomallei* [22]. It is possible that the accessory component of T6SS, TagAB-5, could impact MNGC formation of *B. pseudomallei*. Therefore, we investigated MNGC formation of *B. pseudomallei* Δ*tagAB-5* mutants in HMC3 cells by comparing them with the wild-type K96243 (Figure 5a). At 8 h post-infection, MNGC formation was detected in the HCM3 cells infected by *B. pseudomallei* wild-type K96243, approximately 3.47% ± 0.22% of total counted cells (Figure 5b). On the other hand, the *B. pseudomallei* Δ*tagAB-5* mutant slightly initiated MNGC formation of HCM3 cells (0.43% ± 0.09%), showing a significant difference from the wild-type (*p* = 0.0002) (Figure 5b). Obviously, the ability to form MNGCs could be restored by complementation of the Δ*tagAB-5* mutant (2.73% ± 0.33%), as shown in Figure 5b. This result suggests that TagAB-5 of *B. pseudomallei* is vital for MNGC formation in microglial cells.

### 3.4. TagAB-5 Effects on Damage in B. pseudomallei-Infected HCM3 Cells

**Figure 6:**
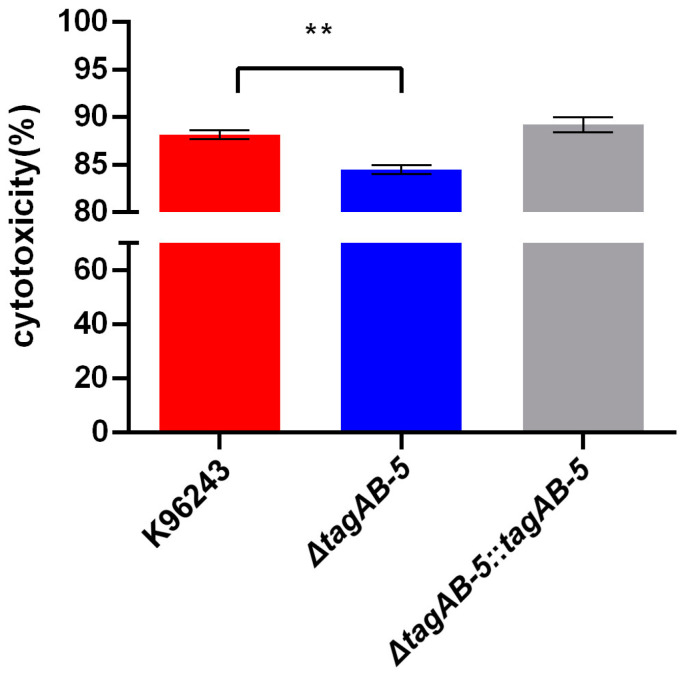
Cytotoxicity of HMC3 cells infected by *B. pseudomallei*. HMC3 cells were infected with *B. pseudomallei* K96243, Δ*tagAB-5*, and Δ*tagAB-5*::*tagAB-5* strains at an MOI of 2 for 10 h. The spontaneous release is the amount of LDH release from the cytoplasm of uninfected cells, whereas the maximum release is the amount released by total lysis of uninfected cells by Triton X-100. Values represent the mean ± SEM from three independent experiments. ** *p* ≤ 0.01 indicates a significant difference compared with wild-type strain K96243.

Most infections mount hostile attacks on host membrane integrity. When the cellular membrane is damaged, a soluble cytoplasmic enzyme, LDH, is released into the extracellular space [31], reflecting host cytotoxicity. Therefore, we investigated whether TagAB-5 can impact cellular damage. At 10 h post-infection, a difference in cellular damage was observed between *B. pseudomallei* wild-type K96243 and *tagAB-5*-deletion mutant (*p* = 0.0050) (Figure 6). The percentage of cytotoxicity of strain K96243 (88.15% ± 0.46%) was significantly higher than that of the *tagAB-5*-deletion mutant (84.47% ± 0.45%). This suggests that TagAB-5 causes damage of *B. pseudomallei*-infected HCM3 cells.

### 3.5. TagAB-5 Strengthens the Inflammatory Response of HMC3 Cells

**Figure 7:**
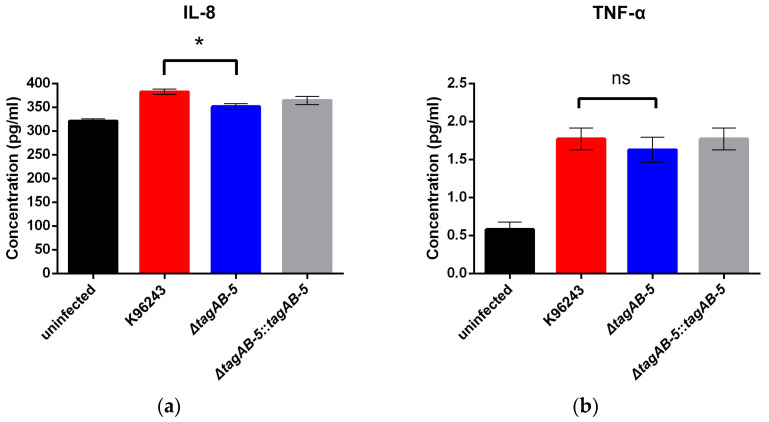
Inflammatory response of HCM3 infected by *B. pseudomallei*. HMC3 cells were infected by *B. pseudomallei* strains with an MOI of 2. The supernatant of HMC3 cells infected with *B. pseudomallei* was collected at 8 h post-infection. Levels of cytokine IL-8 (**a**) and TNF-α (**b**) were determined by ELISA. Data are represented as the means ± SEM. In each independent experiment, there were at least three replicates per group. Statistical significance was determined against the wild-type group using Student’s *t*-test. ns: not significant, * *p* < 0.05.

We found that TagAB-5 is associated with cell damage and MNGC formation of *B. pseudomallei* in human microglial HCM3. We hypothesized that the function of HCM3 in cytokine production might be altered. After infection with *B. pseudomallei*, HCM3 cell culture supernatants were tested with ELISA for IL-8 and TNF-α proteins (Figure 7). Uninfected HCM3 spontaneously produced IL-8 and TNF-α proteins at 321.08 ± 7.70 pg/mL and 0.58 ± 0.17 pg/mL, respectively. Compared to uninfected cells, *B. pseudomallei* K96243 infection stimulated IL-8 and TNF-α at 8 h post-infection with an average production of 382.64 ± 7.37 pg/mL and 1.77 ± 0.20 pg/mL, respectively. Furthermore, the Δ*tagAB-5* mutant increased IL-8 and TNF-α production (351.50 ± 5.88 pg/mL and 1.63 ± 0.17 pg/mL, respectively) compared with those produced from the uninfected cells. The average stimulation of IL-8 and TNF-α by the Δ*tagAB-5* mutant was less than that observed with the wild-type K96243 (Figure 7). However, only HCM3 cells infected with the *B. pseudomallei* Δ*tagAB-5* mutant produced a statistically significant lower amount of IL-8 when compared with K96243-infected cells at 8 h post-infection (*p* = 0.0372).

### 3.6. Role of TagAB-5 in B. pseudomallei Virulence in an In Vivo Model

**Figure 8:**
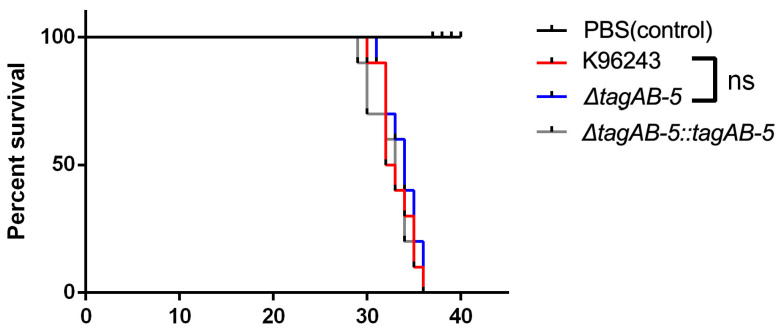
*G. mellonella* larvae were infected with 1 CFU of each strain of *B. pseudomallei* K96243, Δ*tagAB-5*, and Δ*tagAB-5::tagAB-5* strains. The survival of larvae was monitored at 24 h post-infection until to 36 h post-infection. Each data set is representative of a single trial with per-cent survival of infected larvae. Control larvae (PBS-injected larvae) did not die in any given trial. ns: not significant.

We subsequently explored the effect of *tagAB-5* on bacterial virulence using an in vivo *G. mellonella* infection model. Larvae were infected with 1 CFU of *B. pseudomallei* K96243, Δ*tagAB-5*, and Δ*tagAB-5*::*tagAB-5* strains. At 24 h post-infection, larvae survival was monitored (Figure 8). Compared with infected wild-type *G. mellonella*, there was no significant difference observed in Δ*tagAB-5*-infected *G. mellonella*. They exhibited 100% mortality at 36 h post-infection. No mortality was observed in injected PBS larvae. This indicates that *tagAB-5* is not involved in the virulence of *B. pseudomallei* in the *G. mellonella* model.

## 4. Discussion

CNS melioidosis is an important disease threat because it has a high mortality (approximately 20–50%) [8,32]. Therefore, understanding *B. pseudomallei* pathogenicity in establishing CNS infection requires more focus on developing better treatments to minimize health risks of patients with melioidosis with CNS involvement. Several studies employed animal models to investigate how *B. pseudomallei* causes CNS infection. Previously, a monocyte (CD11b+) was found to act as a Trojan horse carrying *B. pseudomallei* across the cerebral endothelium and inducing neurological melioidosis in BALB/c mice [12,20]. Other studies in BALB/C and C75B1/6 mice demonstrated that *B. pseudomallei* cause CNS infection by invading the olfactory nerve or trigeminal nerve [10] and crossing the blood–brain barrier [9,33]. When *B. pseudomallei* uses the nasal route to penetrate into the brain, many cells of the olfactory system and CNS (such as olfactory epithelium, olfactory and trigeminal neurons, glial cells and microglial cells) can be used as intracellular niches for *B. pseudomallei* [9,20]. In this study, we investigated *B. pseudomallei* pathogenesis in human microglia, which are primary innate immune cells that function as macrophages in the CNS, secreting various soluble factors (such as chemoattractants, cytokines, and neurotropic factors) that contribute to various aspects of the immune response and tissue repair in the CNS [34]. Our study employed the human microglial cell line HMC3 for in vitro investigation and found that *B. pseudomallei* clinical strain K96243 was able to invade, survive, and multiply in this cell, which finally led to formation of MNGC. Although using secondary cell culture might show some differences in the response when comparing to the primary cell culture, it has been difficult to study primary cultures of freshly isolated microglia and stem cell-derived microglia since microglia display large overlaps in surface markers to other related myeloid cells [35]. Using HCM3 cell line provides the main advantage as they are immortalized cells, which is the consistency and reproducibility of results that can be obtained from using a batch of clonal cells. Consequently, those results could be indicative the plausible pathogenesis in a host.

The type VI secretion system (T6SS) is a protein delivery machine widely found in Gram-negative bacteria. T6SS is required for killing neighboring cells and pathogens by injecting toxic effectors directly into target cells upon cell-to-cell contact [36]. T6SS are generally composed of core conserved proteins and non-conserved proteins (or accessory proteins) [37]. Based on T6SS nomenclature, the conserved T6SS genes were designated as *tss A-M*, while the accessory genes were designated as *tag A-P* [38]. T6SS is a major virulence determinant of the *B. pseudomallei* in Syrian hamster model [22]. Based on in vivo expression technology, three T6SS-associated genes (*tssH-5*, *tssl-5*, and *tssM-5*) in a cluster of T6SS were induced upon invasion of murine macrophage-like cell line RAW264.7 [38]. Interestingly, one study demonstrated that the T6SS accessory *tagAB-5* (*BPSS1504*) was required for *B. pseudomallei* strain E8 (soil isolate) to survive intracellularly and form MNGC in the macrophage RAW264.7 [21]. Furthermore, TagAB-5 has been shown to be involved with the virulence in the BALB/c mouse model [21]. Similar to an organism closely related to *B. pseudomallei*, *tagAB-5* was up-regulated in the transcriptome of *B. thailandensis* strain E555 during infection of J774A.1 mouse macrophage cells after 6 h post-infection [39]. Although TagAB-5 was required for *Burkholderia* to survive intracellularly in the various murine macrophage cells, the mechanism associated with this process in human macrophages remains unknown.

In this present study, a mutant strain that lacks *tagAB-5* was constructed to further explore the effect of *tagAB-5* deletion on phenotypic and pathogenic characters of *B. pseudomallei* during human microglial HMC3 infection. Our results showed that *tagAB-5* deletion had no significant effect on the in vitro growth of K96243 (Figure 1a). Correspondingly, a previous study of *B. pseudomallei* strain E8 that lacks *tagAB-5* revealed normal growth in LB and minimal Vogel–Bonner medium. Accumulating evidence suggests that TagAB-5 has no influence on growth ability. Bacteria employ protein secrete systems to engage in a variety of processes including nutrient acquisition, motility, competition, and virulence. However, only type VII-specialized secretion systems (T7SSs) have been reported as essential for bacterial physiology [39,40,41]. Several mycobacterial T7SSs (ESX or ESAT-6-systems) are also essential for mycobacterial survival during in vitro growth because they are required for nutrient acquisition and maintaining envelope impermeability [40,41,42].

A previous study showed that mutation of *B. pseudomallei* genes associated with the bacterial membrane (such as LPS and O-polysaccharide modification) can influence colony morphology [43]. T6SS is a secretion nanomachine that is extended through the bacterial double membrane; however, there is no evidence showing its impact on *B. pseudomallei* colony morphology. Because TagAB-5 is a component of T6SS located on the bacterial membrane, it is possible that the lack of this protein could change the colony morphology of *B. pseudomallei*. We tested this phenotype of a *B. pseudomallei* Δ*tagAB-5* mutant compared to the wild-type *B. pseudomallei* K96243 and found that they both have a type I colony morphology. Our results indicate that deletion of *tagAB-5* gene did not affect the colony morphology of this organism and suggest that colony morphology variation is not influenced by TagAB-5.

A recent report found that deletion of the T6SS core component can influence the antimicrobial resistance of *Acinetobacter baumannii* [44]. In this study, we tested the effects of *tagAB-5* deletion on the antimicrobial susceptibility of *B. pseudomallei* with selected seven antibiotics in EUCAST guideline, including amoxicillin-clavulanic acid, ceftazidime, imipenem, meropenem, trimethoprim-sulfamethazole, tetracycline, and chloramphenicol. As shown in Table 3, there were no significant differences of antimicrobial susceptibility profile between wild-type and *tagAB-5* mutant. In accordance with Wang’s finding, there was no effect of mutation of *hcp* genes which encode the T6SS core component hemolysin-coregulated proteins on the antimicrobial resistance of *Salmonella typhimurium* 14028s [45].

Some bacterial proteins related to colony morphology and antibiotic resistance such as an outer membrane protein (OmpA) and chemotaxis protein VCA0893 were seen to be associated with the T6SS genes for the translocation of effector molecules [46]. However, alterations of colony morphology and drug resistance were not found in this study. It is supposable that TagAB-5 might not disturb those proteins. Hence, additional experiments are required to confirm this hypothesis. For example, the expression of OmpA and VCA0893 proteins should be further investigated. Likewise, an interaction network of TagAB-5 should also be further explored to exemplify how this protein involved with known cell morphology and antibiotic resistance-associated proteins. Additionally, structured illumination microscopy (SIM) could be employed to investigate the subcellular localization of T6SS, as demonstrate in *Acinetobacter baylyi* and *B. thailandensis* [47].

Successful establishment of infection by *B. pseudomallei* requires invasion into the host cells. Several virulence factors of *B. pseudomallei* have been reported to facilitate invasion into host cells, such as human lung epithelial cell line A549 [48], human cervical cell line HeLa [49], and human skin fibroblast cell line HFF-1 [50]. A virulence factor of *B. pseudomallei*, cycle-inhibiting factor (Cif), was recently reported to be involved in the invasion into the human neuronal cell line SH-SY5Y [16]. Regarding to the accessory protein of T6SS on ability to invade host cells, the previous study that showed the Δ*tagAB-5* mutant had no effect on invasion of *B. pseudomallei* into the human lung epithelial cell line A549 [21]. This finding agrees with our finding that there is no difference in internalization of the *B. pseudomallei* Δ*tagAB-5* mutant into human HCM3 compared to the wild-type K96243 (Figure 2). It indicates that *tagAB-5* is not involved with the internalization of *B. pseudomallei.* Another possible explanation could be that TagAB-5 is a component of the T6SS that *B. pseudomallei* uses to deliver effectors into target cells. However, these effectors might not be directly involved in internalization.

After bacterial internalization, *B. pseudomallei* replicates in the HCM3 cells (Figure 3a). We found that deletion of *tagAB-5* gene led to a significant defect in the intracellular survival of *B. pseudomallei* at 10 h post-infection in this cell type (Figure 3a). Consistent with the previous study, Δ*tagAB-5* of *B. pseudomallei* strain E8 impaired intracellular survival in the mouse macrophage cell line RAW264.7 and human lung epithelial cell line A549 [21]. Therefore, this suggests that *tagAB-5* is likely to be an essential factor for *B. pseudomallei* intracellular survival. In contrast, although *tssH-5* was identified as a macrophage-inducible gene, the infection assay of the *tssH-5* null mutant showed that TssH-5 is not required for intramacrophage survival of *B. pseudomallei* in RAW264.7 cells [38].

*B. pseudomallei* can hijack the host actin cytoskeleton and promote actin-based motility that leads to cell-to-cell spread [51,52]. Once *B. pseudomallei* survive within the host cytoplasm, the polymerization of actin filament is initiated [53]. Formerly, actin-based motility has been reported to facilitate the invasion into the olfactory nerve during the early stage of *B. pseudomallei* infection [10]. The absence of TagAB-5 has no effect on actin-based motility by *B. pseudomallei* in HCM3 cells (Figure 4). It implies that this protein is nonessential for this behavior. In good agreement with the previous study, induction of actin tail formation within kidney epithelial Ptk2 cells was not affected by deletion of *tagAB-5* gene [21]. Similarly, deletion of *hcp1* (encoding for the core protein of T6SS-1) had no effect on *B. pseudomallei* actin-based motility in murine macrophage-like RAW264.7 [22]. In contrast, *Burkholderia mallei* mutant lacking *tssE* (a core component of T6SS-1) showed defects in actin-based motility and MNGC formation [54]. Based on these findings, it appears that T6SS plays a critical role in actin-based motility of *B. mallei* but not in *B. pseudomallei.*

Cell-to-cell fusion is a unique characteristic of host cells infected by *B. pseudomallei*, which then form MNGC for intracellular spread once sufficient bacterial replication has occurred within an infected cell [52]. The significance of *B. pseudomallei*-mediated MNGC formation during infection is currently unclear. However, it is possible that cell-to-cell spread via MNGC allows the pathogen to evade immune surveillance in vivo. Although MNGC formation of microglia induced by *B. pseudomallei* has never been reported, microglia can form a unique phenotype of MNGC, which are observed in AIDS encephalopathy and tuberculosis [55]. It is well known that T6SS was important for MNGC formation of *B. pseudomallei* [21,22] and *B. mallei* [54]. *B. pseudomallei*, *tagAB-5* and *hcp1* mutants showed impaired MNGC formation in RAW264.7 cells [21]. As expected, we also found that *tagAB-5* plays an important role in MNGC formation in microglia infection. Infected cells initiate cell-to-cell fusion, and few MNGCs were observed in HMC3 cells infected by Δ*tagAB-5* mutant at 8 h post-infection (Figure 5).

The overall result of intracellular survival and MNGC formation by *B. pseudomallei* ultimately leads to cell toxicity or damage. Cytotoxicity is related to the bacterial ability to cause LDH release from the host cells, which is a part of bacterial pathogenicity. Hcp1 is important for *B. pseudomallei* strain K92643 in inducing cell toxicity in the RAW 264.7 murine macrophage cells [22]. Similarly, *tagAB-5* is also crucial for *B. pseudomallei* strain E8 and K96243 in inducing cytotoxicity in C57BL/6 bone marrow-derived macrophages [21] and HMC3 cells (Figure 6), respectively. These findings suggest that *tagAB-5* is involved in the cytotoxicity of various cells, including microglia, infected by *B. pseudomallei*. Consequently, this process might contribute to T6SS-mediated killing of target cells by injecting harmful proteins (effectors), such as pore-forming toxins and nucleases, ultimately leading to host cell lysis [56].

As innate immune cells, microglia function as macrophages within the central nervous system to protect the CNS from pathogens by generating an inflammatory milieu (such as IL-1, IL-6, and TNF-α) [57]. While release of these factors is typically intended to prevent further damage to CNS tissue, they may also be toxic to neurons and other glial cells. In mouse melioidosis with neurological symptoms, pro-inflammatory cytokine levels of IL-6 and TNF-α were relatively high during early infection, but later the level of TNF-α was decreased [13]. This finding suggests that lack of TNF-α induction might cause an incomplete immune response against *B. pseudomallei*. However, there is no evidence showing *B. pseudomallei* provoke the human microglia to induce proinflammatory cytokines.

Proinflammatory cytokines and chemokines have been reported to be involved with meningitis caused by pathogenic bacteria, including *E. coli* and *Haemophilus influenzae* type b [58]. Previous study on immune responses of human fetal microglial cells indicated that the pro-inflammatory chemokine IL-8 was increased in response to bacterial LPS [59]. The main function of IL-8 regarding microglia is the induction of chemotaxis to recruit the immune cells to site of infection and regulation of the inflammatory response. Our investigation into cytokine production during microglia infection with *B. pseudomallei* revealed that stimulating HCM3 cells with wild-type K96243 (MOI of 2) resulted in a slight increase in IL-8 levels at 8 h post-infection compared to uninfected cells. Importantly, the absence of *tagAB-5* causes a statistically significant reduction in the release of IL-8 in HCM3 cells infected with *B. pseudomallei* (Figure 7a). At 8 h post-infection, there is no significant difference in the number of intracellular bacteria (Figure 3a), but different levels of IL-8 can be detected, suggesting the potential involvement of *tagAB-5* in *B. pseudomallei* pathogenicity to elicit the inflammatory reaction. Interestingly, IL-8 was induced in lung epithelial cells A549 infected by *B. pseudomallei* [60]. Moreover, recent research showed that lung epithelial A549 cells treated with vitamin D_3_ exhibited a decrease in a set of cytokines and chemokines including IL-8 along with the reduction in MNGC formation at 12 h post-infection by *B. pseudomallei* [61]. This suggests that the production of IL-8 is likely related to the MNGC formation of A549 cells infected with *B. pseudomallei* [61]. In contrast, TNF-α is not likely related to the MNGC formation of HCM3 cells because there is no difference of TNF-α production between *B. pseudomallei* deficient in *tagAB-5* and possessing *tagAB-5* (Figure 7). Our finding is consistent with a previous study of rat microglia [62]. Treatment with TNF-α failed to induce the MNGC formation in rat microglia, while the addition of interleukin 3, interleukin 4, interferon gamma, and granulocyte–macrophage colony-stimulating factor triggered the formation of MNGC [62]. However, the involvement of TNF-α in the disease severity and fatal outcome of melioidosis has been reported in patients [63] and murine model [64].

The immune system of insects is similar structurally and functionally to the innate immune system of mammals, and thus the results obtained using insects can be applied to mammals [65]. Previously, *G. mellonella* was used to test the virulence of *B. pseudomallei* compared with *B. thailandensis* and *B. oklahomensis*, and the results reflect the virulence observed in murine infection models [66]. *G. mellonella* larvae are widely used as in vivo models to study various pathogens including *Listeria monocytogenes*, a cause of meningitis. Use of developing *G. mellonella* larvae as a model for studying brain infection by *L. monocytogenes* was previously reported [24]. Likewise, the involvement of TagAB-5 in *B. pseudomallei* virulence was tested using *G. mellonella*. Despite the significance of TagAB-5 in the vitro model using human-derived microglia cells, *B. pseudomallei* lacking *tagAB-5* did not affect the survival of *G. mellonella* larvae (Figure 8). Nevertheless, TagAB-5 has been revealed to play an important role in contributing to *B. pseudomallei* virulence in the BALB/c murine model [21]. The diverse outcomes may arise from distinct cellular mechanisms between the two organisms, as well as host-specific factors in *G. mellonella* and human microglia cell models. We suggest that the mouse model may better reflect the relevance to the human microglial cell infection model, and *G. mellonella* infection may not entirely predict mammalian infection. Further comprehensive studies are required to verify significant differences in cellular response mechanisms between insects and mammals.

Until now, CNS melioidosis remains a health threatening disease with a high motility rate due to high virulence of *B. pseudomallei* that is related to specific virulence factors which are crucial for pathogenesis. Although the mechanisms of *B. pseudomallei* pathogenesis are actively studied, the knowledge of the CNS immune cell interactions is limited. Only few virulence factors have been identified to play an essential role in CNS infection. For example, Cif was found to confer the invasion of *B. pseudomallei* into human neuronal SH-SY5Y cells [16]. A recent study revealed that BimA is required for successful intracellular survival and cell fusion upon infection of SH-SY5Y cells [17]. BimA also play the roles in the apoptosis and cytotoxicity of infected neuron cells by *B. pseudomallei* [17]. This present study, we found that TagAB-5 has an important function in intracellular replication, MNGC formation, and induction of IL-8 production in microglia cell. These knowledges are important for identifying the key bacterial proteins as targets for the development of more effective therapeutics for CNS melioidosis, such as production of specific agents targeting TagAB-5 protein or its associated pathways to mitigate the virulence of *B. pseudomallei*. Nonetheless, other virulence factors that contribute to the complications of CNS melioidosis need to be explored. A deeper understanding of the CNS melioidosis’s pathogenesis, particularly the role of virulence factors and host response, may also help to define optimal treatment. This includes exploring alternative therapies and devising strategies to effectively overcome this severe infection.

## 5. Conclusions

CNS melioidosis caused by *B. pseudomallei* has become a severe health threat; nevertheless, little is known about the underlying mechanism of its neuropathogenesis. In this present study, we investigated the role of T6SS accessory protein TagAB-5 in human microglia infection. Our results demonstrated that *B. pseudomallei* harbors TagAB-5 for intracellular survival, MNGC formation, and cytotoxicity induction during HCM3 infection. Moreover, TagAB-5 is important for triggering IL-8 production of HCM3 cells infected with *B. pseudomallei*. These findings provide additional insights into *B. pseudomallei* pathogenicity by highlighting the role of TagAB-5 in neuropathogenesis, which could be considered a potential target for prevention and treatment of severe CNS melioidosis.

## Figures and Tables

**Table 1:** Bacterial strains used in this study.

Bacterial Strains	Description	Source
K96243	*B. pseudomallei* clinical strain	[25]
Δ*tagAB-5*	K96243 *tagAB-5* deletion mutant	This study
Δ*tagAB-5::tagAB-5*	K96243 *tagAB-5* complement strain	This study

**Table 2:** Primers used in this study.

Primer Names	Sequence (5′–3′)	Purpose	Size	Source
F1-BPSS1504	5′-gcgaagcccggggAGCTGAAGGCCAAGCAGA-3′	Amplification of full-length *tagAB-5*	3431	This study
R2-BPSS1504	5′-agcgtccccgggGCGAGGTCGGTTTCCGT-3′			
Seq-F-BPSS1504	5′-CGATGAGCGTCGGCAAGG-3′	Amplification of flanking regions of *tagAB-5*	3566	This study
Seq-R-BPSS1504	5′-CGGCTGAAATGGGTCATCGT-3′			
OriT-F	5′-TCCGCTCATAACCCTGCTTC-3′	Validation of the presence of pEXKm5 plasmid backbone	236	[16]
OriT-R	5′-CAGCCTCGCAGAGCAGGATTC-3′			

Note: Capital letters represent annealing to the targeted DNA whereas lowercase letters are additions to the primer. Underlined letters indicate a recognition site of *Sma*I.

**Table 3:** Effect of *tagAB-5* mutations on the drug susceptibility of antimicrobials.

Strains	AMC	CAZ	IMP	MEM	SXT	TE	C
K96243	I	I	S	S	I	S	S
Δ*tagAB-5*	I	I	S	S	I	S	S
Δ*tagAB-5*::*tagAB-5*	I	I	S	S	I	S	S

AMC: amoxicillin-clavulanic acid, CAZ: ceftazidime, IMP: imipenem, MEM: meropenem, SXT: trimethoprim-sulfamethoxazole, TE: temazepam, and C: chloramphenicol.

## Data Availability

The data presented in this study are available on request from the corresponding author.

## Supplementary Materials

The following supporting information can be downloaded at https://www.mdpi.com/article/10.3390/biomedicines11112927/s1: Figure S1: Plasmid construction for generation of *B. pseudomallei* Δ*tagAB-5* mutant and complementary strains; Figure S2: Effects of MOI on the invasion and replication of *B. pseudomallei* K96243 in HCM3 cells.
