# Supplementary material for: Type VI Secretion System Accessory Protein TagAB-5 Promotes Burkholderia pseudomallei Pathogenicity in Human Microglia
Source: Biomedicines. 2023 Oct 30;11(11):2927. doi: 10.3390/biomedicines11112927 (PMC10669256; doi:10.3390/biomedicines11112927)
Supplement: Supplementary file 1 [file biomedicines-11-02927-s001.zip › biomedicines-2626002-supplementary.pdf]

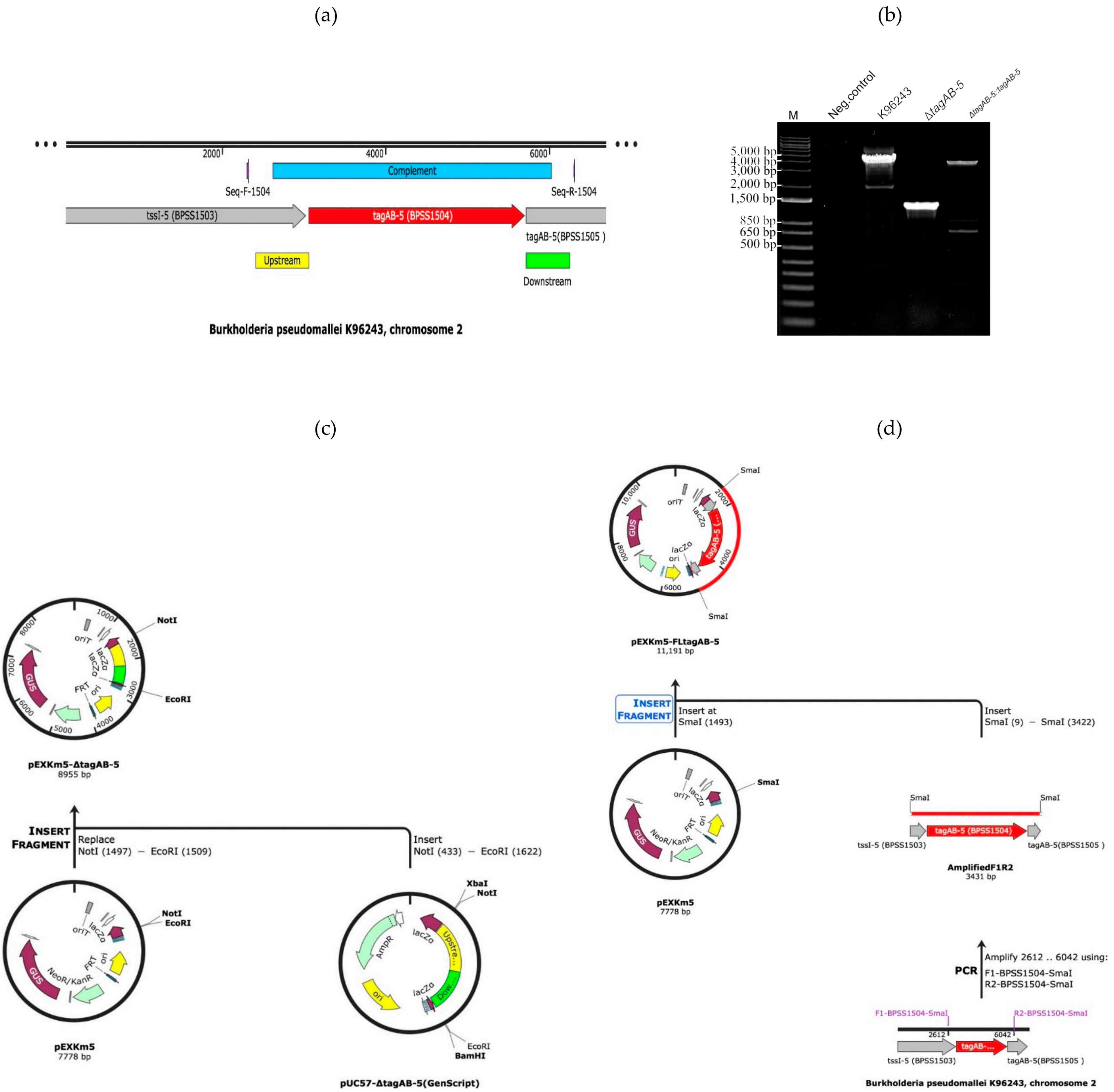

Figure S1. Plasmid construction for generation of *B. pseudomallei* Δ*tagAB-5* mutant and complemented strain. a) Mutation design indicating the regions for gene manupulation. b) PCR product. As expected, a 1,349 bp DNA fragment (lane 4) was detected in *ΔtagAB-5* mutant, whereas a 4,009 bp DNA fragment (lane 3) was detected in the wild-type K96243, indicating successful homologous recombination by deletion of *tagAB-5* gene on the chromosome of *B. pseudomallei* mutant. The amplified product of the complemented strain showed the presence of a 4,009 bp DNA fragment (lane 5), as observed in the parental strain. c) Construction strategy of *B. pseudomallei ΔtagAB-5* mutant. d) Construction of mutagenesis plasmid for generation of *B. pseudomallei ΔtagAB-5:: tagAB-5* complemented strain.

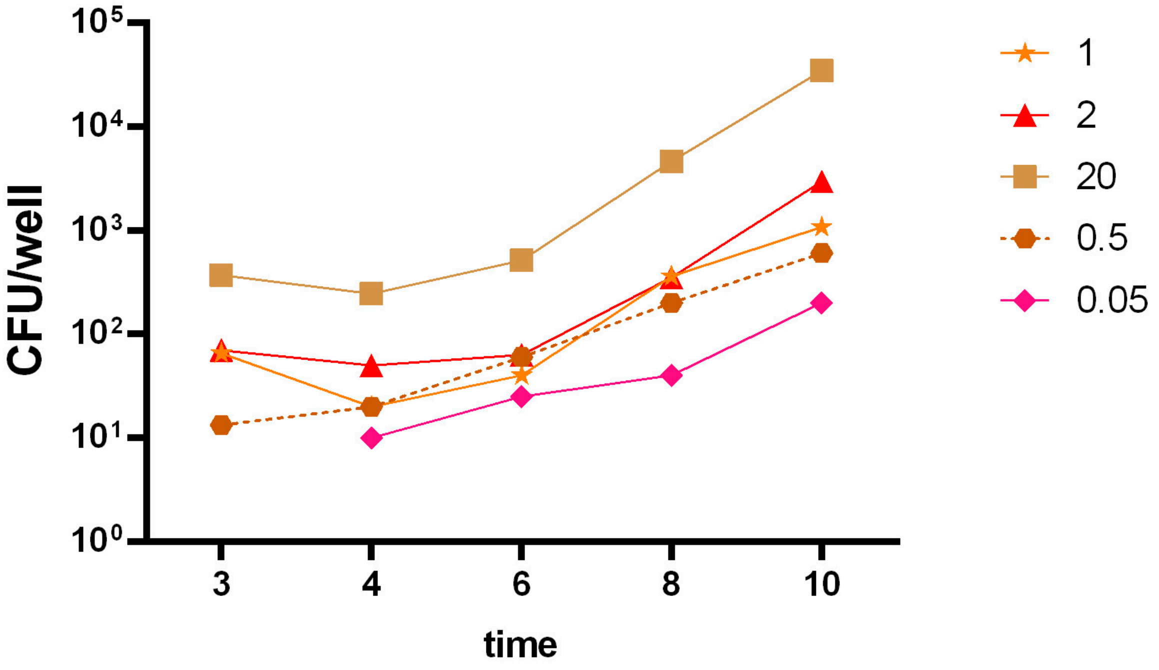

Figure S2. Effects of MOI on the invasion and replication of *B. pseudomallei* K96243 in HCM3 cells. HCM3 cells were infected with *B. pseudomallei* K96243 at MOI of 0.05, 0.5, 1, 2, and 20 for 2 hours. The numbers of intracellular bacteria at 3, 4, 6, 8, and 10 hours post-infection were determined by lysing the infected cells and counting viable intracellular bacteria on culture plates.
